# Homeopathic treatment of patients with chronic sinusitis: A prospective observational study with 8 years follow-up

**DOI:** 10.1186/1472-6815-9-7

**Published:** 2009-07-27

**Authors:** Claudia M Witt, Rainer Lüdtke, Stefan N Willich

**Affiliations:** 1 Institute for Social Medicine, Epidemiology and Health Economics, Charité University Medical Centre, D-10098 Berlin, Germany; 2 Karl and Veronica Carstens-Foundation, Am Deimelsberg 36, D-45276 Essen, Germany

## Abstract

**Background:**

An evaluation of homeopathic treatment and the outcomes in patients suffering from sinusitis for ≥12 weeks in a usual care situation.

**Methods:**

Subgroup analysis including all patients with chronic sinusitis (ICD-9: 473.9; ≥12 weeks duration) of a large prospective multicentre observational study population. Consecutive patients presenting for homeopathic treatment were followed-up for 2 years, and complaint severity, health-related quality of life (QoL), and medication use were regularly recorded. We also present here patient-reported health status 8 years post initial treatment.

**Results:**

The study included 134 adults (mean age 39.8 ± 10.4 years, 76.1% women), treated by 62 physicians. Patients had suffered from chronic sinusitis for 10.7 ± 9.8 years. Almost all patients (97.0%) had previously been treated with conventional medicine. For sinusitis, effect size (effect divided by standard deviation at baseline) of complaint severity was 1.58 (95% CI 1.77; 1.40), 2.15 (2.38; 1.92), and 2.43 (2.68; 2.18) at 3, 12, and 24 months respectively. QoL improved accordingly, with SF-36 changes in physical component score 0.27 (0.15; 0.39), 0.35 (0.19; 0.52), 0.44 (0.23; 0.65) and mental component score 0.66 (0.49; 0.84), 0.71 (0.50; 0.92), 0.65 (0.39; 0.92), 0.74 (0.49; 1.00) at these points. The effects were still present after 8 years with SF-36 physical component score 0.38 (0.10; 0.65) and mental component score 0.74 (0.49; 1.00).

**Conclusion:**

This observational study showed relevant improvements that persisted for 8 years in patients seeking homeopathic treatment because of sinusitis. The extent to which the observed effects are due to the life-style regulation and placebo or context effects associated with the treatment needs clarification in future explanatory studies.

## Background

Chronic sinusitis is generally accepted to be a common illness incurring considerable costs, despite limited epidemiological data[1]. It is defined as an inflammation of the nasal mucosa and paranasal sinuses for at least 12 weeks which may cause nasal blockage or congestion, mucous discharge, facial pain or pressure, and/or impaired smell. Polyps, which may or may not be present are increasingly recognized as part of the sinusitis pathology [1,2]. Several factors have been found to contribute to the disease, namely, insufficient ciliary motility, allergy and asthma, bacterial infection, and more rarely, morphological anomalies, immune deficiencies and Samter's triad (salicylate sensitivity, asthma, nasal polyps). While the role of fungi and hormonal changes during pregnancy are unclear, it may also be an early symptom of systemic disease [1,3,4].

Standard treatment recommendations are to suppress the inflammatory process with corticosteroids [1,5,6], antibiotics may be also necessary to combat opportunistic infections [1,7], and possible underlying diseases may require their own specific medication. Saline douching can provide some symptomatic relief [1,2]. Surgical intervention was found to be as effective as medical treatment, but should be reserved for refractory cases [1,3,5]. Some complementary and alternative medical (CAM) treatments might be helpful as adjuvants [8]. It appears that homeopaths are consulted more frequently by patients with acute and chronic sinusitis (13% of the homeopathy group vs. 7% of the conventional group in an observational comparison study) [9], but to date no research has looked into the effects of homeopathy for chronic sinusitis.

Homeopathy is practised in many regions of the world [10], especially in high-income countries, where it is the most popular treatment form among the traditional, complementary, or alternative medical therapies [10-12]. Homeopathic prescribing accounts for concomitant symptoms in addition to the predominant pathology, therefore the same main diagnosis may be treated with different remedies in different patients ('individualisation'). The prescribed drugs ('remedies') are under constant debate. They are produced by alternating steps of diluting and agitating a starting substance ('potentiating'). After several repetitions, dilutions beyond Avogadro's number are reached, and the probability approaches zero that even a single molecule of the starting substance remains present in the drug. Such 'high potencies' are often used, however their effects are the subject of scientific controversy.

Apparently, the inconsistent results seen in meta-analyses of placebo-controlled trials pooling a great variety of diseases and ailments [13,14] might be a consequence of trial selection [15]. We analyzed the data from our prospective observational study, which globally evaluated details and effects under homeopathic treatment in a usual care situation (3981 patients over 8 years [16-19]) with respect to diagnosis. This paper presents the 134 adults consulting a homeopathic physician because of chronic sinusitis.

## Methods

### Study and Participants

In this prospective multi-centre observational study, patients were included consecutively upon their first consultation with a participating physician, and subsequently followed up, using standardised questionnaires. This paper analyses the patients suffering from sinusitis for ≥12 weeks (defined as "an inflammatory process of the mucous membranes of the paranasal sinuses [resulting] from any condition", ICD-9: 473.9 [20], ICD-10: J32.9) Study physicians were required to have passed certified training in classical homeopathy and have ≥3 years practical experience (details of recruitment: [17]). Written informed consent and approval by the ethics review board of the Charité University Medical Centre were obtained.

### Data Collection

Before treatment (at baseline) and independent of their physicians, patients recorded the complaints that instigated homeopathic treatment, and rated their severity on a numeric rating scale (NRS, 0 = no complaints, 10 = maximum severity) [21]. The health-related quality of life (QoL) was recorded with the MOS SF-36 [22] questionnaire. The first questionnaires were personally given to the patients by the study physicians and were completed before treatment. Patients sent them in sealed envelopes directly to the study office, from where they received follow-up questionnaires after 3, 12, and 24 months, and 8 years, with every complaint being transferred to the follow-up questionnaires to ensure continuous assessment. At baseline, 3, 12 and 24 months, the participating physicians recorded up to 4 diagnoses per patient and assessed their severity on identical NRS. On a continuous basis, they recorded the homeopathic treatment, use of any conventional therapies, and all referrals.

### Statistical Analysis

As outcome measures, we defined: mean sinusitis severity, mean severity of all baseline diagnoses (pooled physician assessment), mean severity of all complaints (pooled patient assessment), and QoL scores. Statistical analysis (using SAS/STAT^© ^v9.2 software) followed the intention-to-treat approach: every included patient entered the final analyses. We replaced missing values as follows: Cured complaints: severity = 0 in subsequent records; deceased patients: severity = 10. The remaining missing values were multiply imputed according to Rubin [23]. Each was given 20 distinct, but plausible values, based on correlations with non-missing values and reflecting the overall variability of data. This generated a total of 20 distinct data tables, each with a full data set. These were analysed separately (see below), and the results pooled to calculate treatment effects and p-values. For each imputed data set, treatment effects were estimated on the basis of a generalised multiple linear regression model, following the recommendations by Diggle et al [24]. We assumed the treatment course to be mixed with a piecewise linear part (0–3 months, 3–24 months, and 24 to 72 months). The serial correlation was assumed to be exponential with time. Standardised effects (d) were calculated by dividing treatment effects as estimated above by baseline standard deviations. They were classified: as |d| > 0.8, large; |d| > 0.5, medium; |d| > 0.2, small.

Usually, patients seek treatment when their health is below average (such as severe pain, low QoL, etc.). A natural alleviation of their diseases (regression to the mean) can be mistaken for an effect at the beginning of treatment [25]. In order to separate regression to the mean and treatment effects, the mean of the target population must be known or plausibly assumed. For the QoL, we applied Mee and Chua's test [26] under the assumption that the patients had the same QoL as the general German population [22]. For the NRS ratings no data describing a normal population is available.

## Results

In the present analysis, we included 134 adult patients (Table 1), who had been suffering from sinusitis for 10.7 ± 9.8 years. These patients were treated by 62 physicians (including 1 Ear, nose and throat (ENT) specialist). Almost all accompanying diagnoses assessed at baseline were chronic diseases that had previously been under treatment-mostly with conventional medicine (Tables 1, 2). All diagnoses seen in more than 5% of the patients were present for at least five years (Table 2). Nasal polyps, immune deficiencies, or fibrosis were not diagnosed.

**Table 1:** Demographics and Baseline Status

**Baseline Population (% & N)**	
Patients Total	100.0% (134)
Female	76.1% (102)
Age (Years, Mean ± SD)	39.8 ± 10.4
≥10 Years School	66.4% (89)
**Patients Expected: Homeopathy... (% & N)**	
- Will Help	67.9% (91)
- Will Maybe Help	30.6% (41)
- Will Not Help	0.7% (1)

**Baseline Diagnoses (Mean ± SD)**	
Total, Number	3.37 ± 0.74
- Severity (NRS)	5.8 ± 1.4
Chronic, Number	3.34 ± 0.76

**Any Baseline Diagnosis Pretreated (% & N)**	
Any Treatment	97.0% (128)
Medication *	87.9% (116)
Surgery	32.6% (43)
Other	65.9% (87)

**8 Year Follow-Up (% & N)**	
Completed Questionnaires	67.9% (91)
Female Responders	79.1% (72)

NRS = Numerical Rating Scale: 10 = maximum, 0 = cured. * Excluding Homeopathy.

**Table 2:** Baseline Diagnoses

	**ICD-10** **(Code)**	**Patients** **(% & N)**	**Severity** **(NRS)**	**Duration** **(Years)**
Chronic Sinusitis	J32.9	100.0% (134)	5.9 ± 1.7	10.7 ± 9.8
Eczema	L30.9	9.7% (13)	4.2 ± 1.8	5.7 ± 5.6
Chronic Bronchitis	J42	8.2% (11)	6.3 ± 1.9	7.6 ± 7.2
Headache	R51	8.2% (11)	4.5 ± 1.4	10.8 ± 12.6
Allergic Rhinitis	J30.4	8.2% (11)	5.8 ± 1.4	14.3 ± 11.5
Dysmenorrhoea	N94.6	7.5% (10)	6.9 ± 1.3	15.4 ± 9.6
Migraine	G43.9	7.5% (10)	6.6 ± 1.3	10.3 ± 9.6
Asthma	J45.9	7.5% (10)	5.7 ± 2.6	15.8 ± 13.1
Frequent Infections	R68.8	6.7% (9)	6.9 ± 1.1	5.8 ± 2.8
Depression	F32.9	5.2% (7)	5.7 ± 2.6	12.3 ± 14.4
Gastritis	K29.5	5.2% (7)	5.1 ± 1.2	6.3 ± 4.7
Fatigue	R53	5.2% (7)	6.7 ± 2.3	5.8 ± 5.8

NRS = Numerical Rating Scale: 10 = maximum, 0 = cured. Only diagnoses seen in ≥5% of the patients.

The consultations consisted of an extensive initial consultation (table 3), followed by the analysis of the case. Almost all patients received the first homeopathic medication on the day of their first consultation, three patients had to wait for ≤1 week, ≤1 month, and longer, respectively. The subsequent consultations, about half of them telephone calls, were much shorter than the initial history taking (Table 3). Almost 60% of the patients were still in homeopathic care or had only suspended it temporarily at 24 months (32% after 8 years) (table 3).

**Table 3:** Consultations and Continuance

**Consultations During Study (Mean ± SD)**	
1st Consultation (min)	126 ± 39
Case Analysis (min)	46 ± 47
Follow-up Number, All	9.1 ± 10.2
- Telephone	4.6 ± 7.7
- Practice	3.6 ± 4.3
Follow-up Duration (min), All	22.8 ± 14.5
- Telephone	7.0 ± 4.7
- Practice	34.6 ± 15.6
Follow-up Cumulated (min), All	231.4 ± 161.2
- Telephone	55.1 ± 59.9
- Practice	180.7 ± 119.9
Last Consultation (Month)	17.3 ± 10.1
**Homeopathy At Study End (% & N)**	
Treatment Ongoing	37.3% (50)
Changed Homeopath	1.5% (2)
Currently Not Treated	20.1% (27)
Ended because of...	
- Cure or Amelioration	6.0% (8)
- Reason Outcome-Unrelated	4.5% (6)
- No Effect or Aggravation	11.9% (16)
- Not Stated Reason	0.7% (1)
No Answer to Treatment Status	17.9% (24)

**Homeopathy At 8 Year Follow-Up (% & N)**	
Under Treatment	18.7% (25)
Changed Homeopath	13.4% (18)
Ended because of...	
- Cure or Amelioration	10.4% (14)
- Reason Outcome-Unrelated	6.0% (8)
- No Effect or Aggravation	16.4% (22)
- Not Stated Reason	2.2% (3)
No Answer to Treatment Status	32.8% (44) *

* Including not returned questionnaires.

**Figure 1:**
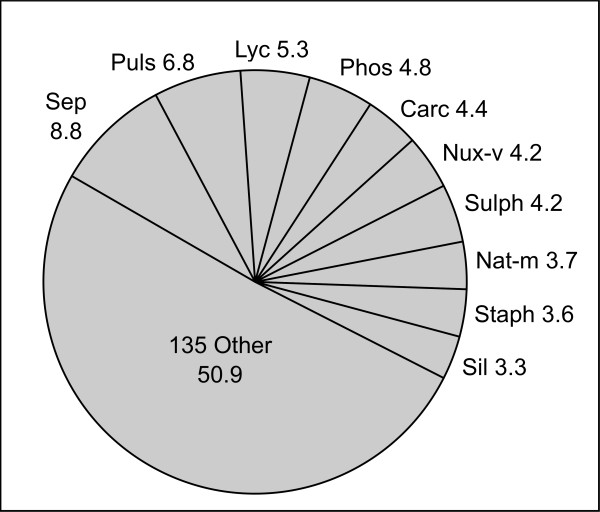
**Most Frequently Prescribed Homeopathic Remedies (after 24 Months)**. Percent of prescriptions during study period, remedies identified with traditional abbreviations (in decreasing order of frequency: Sepia, Pulsatilla, Lycopodium, Phosphorus, Carcinosinum, Nux vomica, Sulphur, Natrium muriaticum, Staphisagria, Silicea).

In the first 24 months, patients received 8.3 ± 6.2 homeopathic prescriptions. Half of all prescriptions were covered by 10 homeopathic remedies (figure 1), but in total, 145 remedies were applied. Most used were the potencies: C200, 35.7%; C1000, 23.0%; C30, 14.2%; C10000, 7.6%; Q1, 3.5%; D12, 3.3%. (Letters indicate dilutions steps during manufacturing: 1/100 for centesimal (C-) potencies, 1/50000 for quinquagintamillesimal (Q-) potencies, and 1/10 for decimal (D-) potencies; numbers give the step repetitions. For example, a "C200" preparation is diluted-1/100-then-agitated 200 times. Thus, 88.3% of the remedies were potentiated to a dilution beyond Avogadro's number.

The strongest improvements in the severity of complaints were seen in the first 3 months, they generally continued during the first 24 months and persisted for another 6 years (Table 4). The physicians' assessments of the severity change tended to be more positive than patients' own assessments (data not shown). The improvements in health-related QoL were of smaller effect size (Table 4) but statistically significant. The latter was confirmed by Mee-Chua-tests for the mental component score (p = 0.0859, p = 0.034, and p < 0.0001 at 3, 12 and 24 months) but not for the physical component score (p = 0.6869, p = 0.6859, and p = 0.1259). After 24 months, sinusitis and other baseline diagnoses were considerably relieved (Table 5), while large reductions in the use of conventional medicines and health care services were observed (Table 6). The observed improvements were still present in the 8-year follow-up (Table 4).

**Table 4:** Diagnoses, Complaints, Quality of Life

***Status***	**Baseline**	**Month 3**	**Month 12**	**Month 24**	**Year 8**
**Severity (NRS)**					
Sinusitis ‡	5.88 (5.55; 6.22)	3.11 (2.78; 3.44)	2.11 (1.78; 2.44)	1.63 (1.29; 1.96)	--
Pooled Diagnoses ‡	5.80 (5.50; 6.11)	3.63 (3.32; 3.93)	2.66 (2.36; 2.96)	2.06 (1.75; 2.36)	--
Pooled Complaints †	6.03 (5.70; 6.35)	3.57 (3.22; 3.91)	3.12 (2.86; 3.38)	2.80 (2.52; 3.09)	2.49 (2.13; 2.85)
**Quality of Life (SF-36 Component Scores)**					
Physical †	45.60 (43.42; 47.77)	49.02 (46.75; 51.29)	50.06 (48.09; 52.02)	51.12 (49.08; 53.16)	50.52 (47.97; 53.08)
Mental †	37.08 (35.01; 39.15)	43.96 (41.75; 46.16)	44.43 (42.68; 46.18)	43.86 (41.98; 45.74)	46.92 (44.46; 49.37)

***Change***		**Months 0–3**	**Months 0–12**	**Months 0–24**	**Month 0-Year 8**
**Severity (NRS)**					
Sinusitis ‡	--	-2.77 (-3.10; -2.45)	-3.77 (-4.17; -3.37)	-4.25 (-4.69; -3.82)	--
Pooled Diagnoses ‡	--	-2.18 (-2.41; -1.94)	-3.14 (-3.45; -2.84)	-3.75 (-4.09; -3.40)	--
Pooled Complaints †	--	-2.46 (-2.82; -2.11)	-2.91 (-3.28; -2.54)	-3.22 (-3.66; -2.78)	-3.49 (-3.97; -3.02)
**Quality of Life (SF-36 Component Scores)**					
Physical †	--	3.43 (1.90; 4.95)	4.46 (2.38; 6.54)	5.53 (2.84; 8.21)	4.74** (1.31; 8.16)
Mental †	--	6.88 (5.03; 8.72)	7.35 (5.13; 9.57)	6.78 (4.03; 9.53)	9.36 (6.11; 12.62)

Effect Size *		**Months 0–3**	**Months 0–12**	**Months 0–24**	**Month 0-Year 8**
**Severity (NRS)**					
Sinusitis ‡	--	1.58 (1.77; 1.40)	2.15 (2.38; 1.92)	2.43 (2.68; 2.18)	--
Pooled Diagnoses ‡	--	1.56 (1.72; 1.39)	2.25 (2.47; 2.03)	2.68 (2.93; 2.43)	--
Pooled Complaints †	--	1.52 (1.74; 1.30)	1.80 (2.03; 1.57)	1.99 (2.26; 1.72)	2.16 (2.45; 1.86)
**Quality of Life (SF-36 Component Scores)**					
Physical †	--	0.27 (0.15; 0.39)	0.35 (0.19; 0.52)	0.44 (0.23; 0.65)	0.38** (0.10; 0.65)
Mental †	--	0.66 (0.49; 0.84)	0.71 (0.50; 0.92)	0.65 (0.39; 0.92)	0.74 (0.49; 1.00)

Estimated means and 95% confidence intervals from the statistical model (see text). * Absolute value of Cohen's d, usually classified as |d| > 0.8, large; |d| > 0.5, medium; |d| > 0.2, small. ** p < 0,01, all other non-baseline values p < 0.001. † Patients' answers, ‡ physicians' answers. NRS = Numerical Rating Scale: 10 = maximum, 0 = cured. SF-36 = 36-Item Short Form Survey Instrument, higher values = better.

**Table 5:** Response Rates at Study End

**Responders, Chronic Sinusitis (Patients, % & N)**	
Fully Cured	31.3% (42)
Better by ≥50% Baseline	22.4% (30)
Better than 10% but <50%	5.2% (7)
Change within ± 10%	0.7% (1)
Worse >10%	0.7% (1)
**Responders, All Diagnoses (Diagnoses, % & N)**	
Total	100.0% (335)
Fully Cured	33.4% (112)
Better by ≥50% Baseline	29.0% (97)
Better than 10% but <50%	7.5% (25)
Change within ± 10%	4.5% (15)
Worse >10%	1.5% (5)

**Table 6:** Use of Other Treatment and Health Care Services during Study (24 Months)

	**Baseline**	**3 Months**	**12 Months**	**24 Months**
Patients Using Conventional Drugs (% & N) †*				
Any Drug	54.5% (73)	31.3% (42)	34.3% (46)	33.6% (45)
ATC-Class J-Systemic Anti-Infectives	6.0% (8)	1.5% (2)	0.7% (1)	0.7% (1)
ATC-Class R-Respiratory system	37.3% (50)	18.7% (25)	10.4% (14)	9.7% (13)
Antibiotics	4.5% (6)	0.7% (1)	0.7% (1)	0% (0)
Corticosteroids	1.5% (2)	0.0% (0)	0.7% (1)	0% (0)
Patients Using Nonpharmaceutical Treatments (% & N) †*				
Any Therapy	71.6% (96)	20.9% (28)	35.1% (47)	43.3% (58)
Surgery	32.1% (43)	3.0% (4)	9.0% (12)	14.2% (19)
Non-Surgical	64.2% (86)	19.4% (26)	31.3% (42)	39.6% (53)
Acupuncture	25.4% (34)	3.0% (4)	6.7% (9)	9.0% (12)
Patients Consulting Other Health Care (% & N) †*				
Any Physician	98.5% (132)	41.0% (55)	65.7% (88)	77.6% (104)
General Practitioner	71.6% (96)	11.9% (16)	23.9% (32)	36.6% (49)
ENT-specialist	67.9% (91)	10.4% (14)	18.7% (25)	25.4% (34)
Allergy specialist	0.7% (1)	0.0% (0)	0.0% (0)	0.7% (1)
Pulmonary specialist	7.5% (10)	2.2% (3)	3.7% (5)	5.2% (7)
Surgery	5.2% (7)	0.7% (1)	3.0% (4)	5.2% (7)
Hospital	25.4% (34)	2.2% (3)	9.7% (13)	12.7% (17)
Any CAM Treatment	25.4% (34)	0.7% (1)	5.2% (7)	10.4% (14)
Other Homeopath	20.9% (28)	0.7% (1)	2.2% (3)	5.2% (7)
Non-medical CAM Practitioner	3.7% (5)	0.0% (0)	2.2% (3)	3.0% (4)
		**Months 1–3**	**Months 4–12**	**Months 12–24**
**Referrals By Study Homeopath (Patients, % & N) ‡**				
Any Physician *	--	0.0% (0)	0.7% (1)	3.0% (4)
Hospital, Surgery	--	0.0% (0)	0.7% (1)	0.7% (1)

Multiple answers possible. ^† ^Patients' answers, ^‡ ^physicians' answers. * Including all diagnoses/complaints and routine checks (e.g., dentist, gynaecologist). ATC = Anatomical Therapeutic Chemical Classification System.

## Discussion

This prospective multicentre observational study was aimed to provide an overview of contemporary homeopathic health care and the outcomes in 134 patients with chronic sinusitis. During the observation period, assessments of disease severity and health-related quality of life (QoL) consistently showed substantial improvements, although the disease was long-standing, and had previously been treated with conventional medicine. Similarly, the accompanying diseases (almost all chronic) were markedly ameliorated. Although the major improvements took place within the first 3 months of homeopathic treatment, they were still seen after 8 years. Accordingly, QoL increased and use of health care services or conventional medication decreased markedly.

The methodological strengths of our study include the consecutive patient enrolment and use of standardised outcome instruments. The participation of about 1% of all certified homeopathic physicians in Germany (representing 14% of the members of an association for physicians practising 'classical' homeopathy, the Hahnemann Association) in the main study makes the study and the subgroup presented in this paper a reasonably representative sample for contemporary homeopathic practice. We decided against a random sample of homeopathic physicians but recruited physicians trained and certified in 'classical' homeopathy, the type of homeopathy that is accepted and certified by the German Medical Association. Therefore our results are only representative for this type of homeopathy.

In contrast to randomised trials, our study describes patients from everyday practice with multiple morbidities and varying lifestyles. This ensures a high degree of external validity that allows extrapolation to usual medical care. The study, which was designed to evaluate homeopathic treatment of patients suffering from various diagnoses, could not use disease-specific instruments. We decided on a numeric rating scale which is validated, often used [21] and also accepted to measure pain. In addition, we used generic QoL questionnaires.

In this analysis we included patients who had been suffering from sinusitis for ≥3 months in order to approximate most closely the current definition of chronic sinusitis [1,3] with the available data. A shorter duration (e.g., 8 weeks [5]) would have resulted in a but less clearly defined population (+20 patients). In future research, assessments and diagnoses by ENT-specialists would be valuable, to ensure the diagnosis through more standardised and objective criteria. Given the baseline data we can safely assume that almost all patients had been diagnosed with sinusitis by one or more physicians, before the study began.

The majority of the patients were burdened with multiple chronic diseases (like the population of other investigations [27-29]), some of which are among the most frequent illnesses observed in other homeopathic observational studies [29,30]. Several factors could shift the selection towards patients with chronic diseases. As a general observation (especially for industrialised countries) homeopathy patients tend to be younger and better educated than conventional patients, of higher socioeconomic status, and are more often female [31]. These factors could be indicative of increased health-awareness and an inclination toward self-treatment for lesser ailments [32]. Waiting list time of up to several months can be longer than the acute illness itself that might have initiated homeopathic treatment, leaving only chronic diseases as initial diagnoses. The reputation of homeopathy as a 'medicine for the whole person' (reflected in the extensive initial history taking) may cause a self-selection of patients seeking more than a quick fix for a single issue. Finally, the long duration of the diseases (also observed elsewhere [27,30,33]) together with the high rate of previously treated patients, could indicate that most patients turn to homeopathy after finding conventional care unsatisfactory for their conditions. It would be interesting to track an unselected patient cohort through various self-chosen treatments and to do a combined analysis of health status, QoL, and costs. The cost-effectiveness of homeopathic treatment has not been thoroughly investigated so far [34,35]. Medication costs are negligible, while the duration of homeopathic consultations (Table 3) is clearly longer than the 7.6 ± 4.3 minutes of a German GP consultation [36]. This might be compensated by their low frequency. (Conventional consultations take place about 24 times per patient over a 24 month period with a resulting doctor workload of about 190 min in two years [37].)

All estimated health effects were large. This could be mainly explained by placebo and context effects as well as regression to the mean, that our study was not designed to control (effects in between-group comparisons are usually smaller). Nor can we rule out an overestimation of the effect. That the patients' ratings had decreased somewhat at the follow-up may reflect 8 years of ageing, or the wearing off of a novelty effect added to the treatment effect that had caused a possible initial overestimation.

The observed QoL improvements can hardly be caused by regression toward the mean. Assuming chronically ill patients with often several severe diseases to have the same QoL as the general German population was itself a rather conservative approach. Also, patients received homeopathic treatment after years of other treatment and a waiting period – it is very likely that regression toward the mean would have taken place before the first QoL (and NRS) ratings. The same applies to the response shift (patients change internal standards, values, and their QoL concept in reaction to health status changes) [38], which is also likely to shift ratings towards an underestimation of effects.

Our study evaluated the complete package of homeopathic treatment, including context and placebo effects and possible additional treatments in a usual care situation. The extent to which the observed effects are due to the applied homeopathic remedies cannot be determined because no suitable methodology was used. Therefore our study must not be interpreted to support conclusions regarding the efficacy of homeopathic remedies in sinusitis treatment, but rather the total effect of consulting a homeopath. We were also unable to find other evaluations of high-potency homeopathy for chronic sinusitis (one study [9] included any sinusitis but pooled all diagnoses), so the question of remedy efficacy remains unanswered.

It is unlikely that the observed reduction in conventional or alternative medication and treatments are due only to the improved health condition. The homeopathic strategy to reduce interventions to a minimum (which makes classical homeopaths effective 'gatekeepers') is also reflected here for sinusitis, this includes the use of decongestants. In addition, other drugs, stimulating agents, remedy specific 'antidotes', or behaviours that cause known individual aggravations are usually controlled [39]. The type of classical 'homeopathic treatment' investigated in the present study includes a certain amount of lifestyle regulation and health education that most likely contribute to the outcome, as do placebo and context effects. Inactive treatments have strong effects on neuroimmune responses [40] that are likely to affect an inflammatory disease such as sinusitis. Other aspects of treatments (their 'context') may trigger the same mechanisms and they might be more influential than currently acknowledged. For example, the expectations of the patients and the convictions of the physicians regarding the effects and effectiveness of the treatment could be powerful response triggers [40,41]. Both are of course in concordance with the medical approach or philosophy of the respective therapy (for homeopathy, see [42,43]). This makes patients' self-selection into treatment courses a valuable contribution to healing. More generally speaking, every distinct treatment will attract a population that reacts to it [44]. Besides the debated effect of homeopathic remedies, the patients in our study are likely to have profited from the way homeopathy is perceived socially and psychologically. Interestingly, theory and practice of homeopathy have in its history gone through several modifications that (unintentionally) increased the non-pharmacological active factors (e.g., longer and more detailed consultations, increased attention to psycho-social issues, conceptual bridges to the outlook of local cultures and attitudes) [45]. The true extent of placebo/context effects in homeopathic treatment has not yet been investigated, and disentangling the above factors will be a challenging but promising task for future research. Further research in to everyday homeopathic practice, may yield insights into curative means that can be augmented in other areas of medicine [46-49], thus improving health care with respect to health economics and patient benefit.

## Conclusion

Patients with sinusitis treated with 'classical' homeopathy showed marked health and quality of life improvements that lasted for 8 years. The extent to which the observed effects are due to lifestyle regulation and placebo or context effects associated with the treatment needs clarification in the future from more explanatory studies.

## Abbreviations

WHO: World Health Organization; ICD: International Classification of Diseases; NRS: Numerical Rating Scale; QoL: health-related Quality of Life; MOS SF-36: Medical Outcomes Trust 36-Item Short Form Survey Instrument; KINDL: KINDer Lebensqualitätsfragebogen; C*n*: *n*th Centesimal potency; Q*n*: *n*th Quinquagintamillesimal potency; GP: General Practitioner; RCT: Randomized Controlled Trial; ENT-specialist: Ear, Nose and Throat specialist

## Competing interests

This work was supported by a grant from the Karl und Veronica Carstens-Foundation, D-Essen, for SNW and CMW. All authors had full access to all the data in the study and take responsibility for the integrity of the data and the accuracy of the data analysis. Conflicts of interest: None.

## Authors' contributions

CMW conceived and designed the study, interpreted the data, drafted and revised the article. RL designed the study, analysed the data (statistics), revised and approved the article. SNW secured funding, designed the study, revised and approved the article. All authors read and approved the final manuscript.

## Pre-publication history

The pre-publication history for this paper can be accessed here:


